# Molecular transport and water condensation inside mesopores with wettability step gradients†

**DOI:** 10.1039/d3na00594a

**Published:** 2023-10-03

**Authors:** Laura Despot, Chirag Hinduja, Robert Lehn, Joanna Mikolei, Timo Richter, Kilian Köbschall, Mathias Stanzel, Rüdiger Berger, Jeanette Hussong, Marcelo Ceolín, Annette Andrieu-Brunsen

**Affiliations:** a Ernst-Berl-Institut für Technische und Makromolekulare Chemie, Technische Universität Darmstadt 64289 Darmstadt Germany annette.andrieu-brunsen@tu-darmstadt.de; b Max Planck Institute for Polymer Research Ackermannweg 10 55128 Mainz Germany; c Institut für Strömungslehre und Aerodynamik, Technische Universität Darmstadt Peter-Grünberg-Straße 10 64289 Darmstadt Germany; d Instituto de Investigaciones Fisicoquímicas Teóricas y Aplicadas, Universidad Nacional de La Plata and CONICET Diag. 113 y 64 (1900) La Plata Argentina

## Abstract

The wettabilities of nanoscale porous surfaces play important roles in the context of molecular and fluid transport or oil–water separation. The wettability pattern along a nanopore strongly influences fluid distribution throughout the membrane. Mesoporous silica thin films with gradually adjusted wettabilities were fabricated *via* cocondensation. With consecutive mesoporous layer depositions, double-layer mesoporous silica films with asymmetric or so-called Janus wettability patterns were generated. The effects of these wetting gradients on mass transport, water imbibition, and water vapor condensation were investigated with ellipsometry, cyclic voltammetry (CV), drop friction force instrument (DoFFIs), fluorescence microscopy and interferometry. By increasing the film thickness of the hydrophobic mesoporous silica top layer deposited on a hydrophilic mesoporous silica layer up to 205 nm, molecular transport through both the layers was prevented. However, water was observed to condense onto the bottom layer, and transport occurred for thinner top layers.

## Abstract 2

Mesoporous silica films with adjustable wettability and wettability step gradients are fabricated. An influence of the upper hydrophobic layer thickness on water vapor adsorption and condensation as well as on mass and fluid transport is observed.

## Graphical abstract

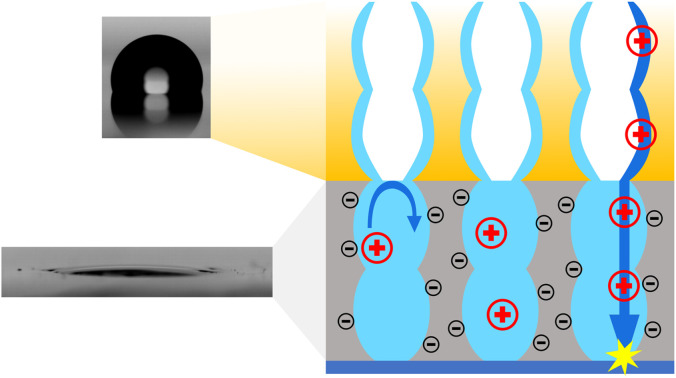

## Introduction

The wettabilities of nanochannels and understanding of their effects on mass transport are of interest in technical applications such as energy conversion^1,2^ or separation processes.^3,4^ This includes selective and directional mass transport.^5^ Modulating and regulating transport properties have been intensively studied with synthetic nanopores and nanochannels.^6–8^ To generate an intrinsic driving force and thus a directional flow, an asymmetric design of the separation layer is needed, which can lead to preferred transport directions for mass and charge, as is seen with biological pores and channels.^9,10^ Conical nanopores as well as Janus-type membranes inherit asymmetric properties such as asymmetric geometries or wettabilities.^11,12^ A preferential direction for ion flow through conical nanopores, which resulted in enhanced ion-current rectification compared to cylindrical pores, was demonstrated by Siwy and coworkers.^13,14^ In a study by Tufani *et al.*, an aluminum oxide membrane was coated with poly(methylacrylic acid-*co*-ethylene glycol dimethacrylate) and poly(4-vinylpyridine-*co*-ethylene glycol dimethacrylate) by chemical vapor deposition to create a membrane with different wettabilities on opposing sides.^15^ This Janus membrane was used for gating the transport of a model protein, bovine serum albumin, by blocking and releasing the molecules in response to changes in the pH of the surrounding solution. Another approach for modifying inorganic materials to generate Janus-type properties for oil–water separation was shown by Cheng *et al.*,^16^ who performed side selective functionalization of a copper mesh with a fluoroalkyl silane to achieve unidirectional water transport. Water did not permeate from the hydrophilic to the hydrophobic side but permeated through the opposite direction, rendering the material a “water-diode”.^17^ Side-selective oil-water separation was also achieved by Herzog *et al.*, who used Janus lab-engineered eucalyptus sulfate paper sheets functionalized with tetraethoxysilane (TEOS) *via* dip-coating.^18^ Tian *et al.*^19^ incorporated wetting gradients into their simulations by using a model composed of spaced microcylinders with a wettability gradient along the thickness and thereby improved the critical breakthrough pressure with the spacing ratio of the cylinder membrane and by increasing the wettability gradient. The mechanism for unidirectional fluid transport was studied by Si *et al.*,^20^ who used a microporous nickel foam with asymmetric wettability. Miao *et al.*^21^ investigated water transport through a three-layer fabric with a continuous wetting gradient. They showed that a transition layer accelerated water transport. Nevertheless, mechanistic investigations were only carried out on the micrometer scale.

Simulations showed that distributions of binary fluid mixtures with different polarities in a nanochannel was influenced by pore wall interactions. After imbibition into the nanochannel, the pore-wall wetting fluid was reported to form a monolayer on the surface, resulting in a fluid front that was enriched in the fluid with the greater polarity difference compared to that of the nanochannel walls. After leaving the pore, the fluids were fully remixing.^22^ The influence of wetting gradients along nanoscale pores was also shown with mesoporous thin films. For example, Lin *et al.*^23^ used a two-layer film consisting of one hydrophilic mesoporous silica layer and an ∼2 nm thick hydrophobic PDMS top layer. By applying a voltage to this double-layer film, the pH changes induced silanol group deprotonation, resulting in an electrostatic force, that overcame the hydrophobic barrier of the thin top layer. Interestingly, the effect of the hydrophobic top layer thickness was not considered. In addition to a few studies on mass transport in porous materials with wettability gradients in mesoporous multilayers, Fuertes *et al.*^24^ investigated water vapor adsorption into ceramic multilayer mesoporous films. They showed that the solvent vapor sorption behavior of a mesoporous oxide layer within a multilayer differed from that of an isolated film. Solvent vapor sorption into a specific layer of a multilayer mesoporous film was influenced by the neighboring layers. Angelomé *et al.* further developed this approach for Tamm mode-based devices that were used as vapor sensors.^25^

Nevertheless, the effects of wetting gradients and water vapor condensation on water imbibition and mass transport were not systematically investigated. The effects of wetting gradients in mesoporous films on water imbibition and condensation and molecular transport and the thickness of a hydrophobic top layer remain open research questions.

Here, we fabricated single-layer silica films with gradually adjustable wettabilities *via* cocondensation of TEOS, triethoxymethylsilane (TEMS) and diethoxydimethylsilane (DEDMS), followed by fabrication of double-layer mesoporous silica films with asymmetric wettabilities and precisely adjusted nanoscale thicknesses and nanoscale pore sizes. Cocondensation of TEOS and TEMS has been used by Guillemin *et al.*^26^ to modulate mass transport through a mesoporous silica thin film. Hayase *et al.*^27^ cocondensed TEMS and DEDMS to fabricate materials for oil–water separation. More recently, the cocondensation of TEOS and TEMS has been used to obtain hybrid silica films for antireflection coatings.^28^ Wettability-dependent water vapor adsorption and molecular transport were investigated with ellipsometry, CV, fluorescence microscopy and interferometry. In addition, drop friction force measurements^29^ was performed to study the influence of water imbibition on the friction of a water droplet located at the outer surface of the mesoporous silica film. An effect of the bottom layer charge, as well as the top layer thickness, on the water vapor condensation and molecular transport was observed with CV.

## Results

**Fig. 1:**
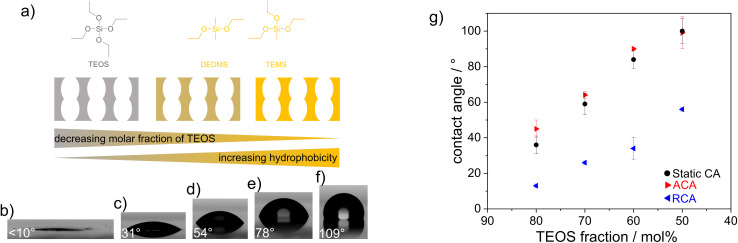
(a) Silica precursors used for cocondensation and tuning the wettability of resulting silica films. Contact angle images of silica films with TEOS molar fraction of 100 (b), 80 (c), 70 (d), 60 (e) and 50 mol% (f). (g) Influence of the TEOS fraction on the macroscopic contact angle.

In general, condensation of TEOS leads to an inorganic, hydrophilic material, whereas condensation of TEMS and DEDMS results in hydrophobic, methylated materials. By combining TEOS, TEMS and DEDMS (Fig. 1a), silica films with adjustable wettabilities were fabricated *via* cocondensation, sol–gel chemistry, and well-established evaporation-induced self-assembly.^30^ The use of cocondensation instead of post-functionalization has the advantage of allowing subsequent multilayer formation with adjustable composition and thus adjustable wettability. Decreasing the molar fraction of TEOS in the sol–gel precursor solution from 100 mol% to 50 mol% gradually increased the hydrophobicities of silica films with advancing water contact angles (ACAs) of up to 100° (Fig. 1f and g). As the mesoporous films were relatively thin and were substrate supported, the volume of water imbibing into the silica film was lower than the drop volume. Thus, the surface hydrophobicity was still reflected in the CA measurements for ACA <90°. This led to the dependence of the ACA on the film thickness, as imbibition occurred only in a ring-shaped area around the droplet due to evaporation, as reported in the literature.^31–33^

**Fig. 2:**
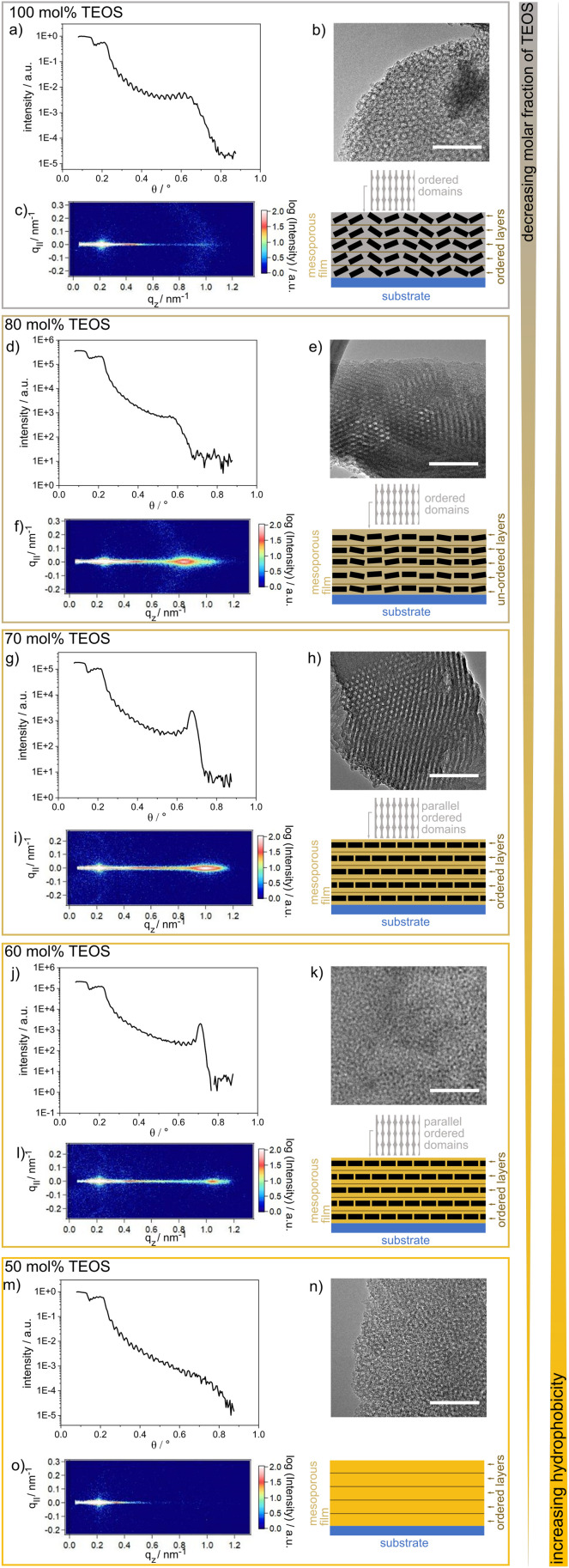
(a), (d), (g), (j) and (m) Results of the XRR experiments on silica wafers coated with mesoporous silica films containing different TEOS molar ratios. Except for the mesoporous silica film with a TEOS molar fraction of 80 mol%, the XRR measurements for the mesoporous silica films show fringes which are typical for ordered layers inside the film. The GISAXS data in (f), (i) and (l) show patterns indicating domains oriented parallel to the substrate, whereas the pattern in (f) starts to vanish and a halo occurs. Halo formation was ascribed to loss of the parallel orientation of the domains. In (c) only, a halo and no pattern is observed and in (o) no pattern or halo is detected. In addition, TEM images of silica films with a TEOS molar fraction of 100 (b), 80 (e), 70 (h), 60 (k) and 50 mol% (n) are shown. Scale bar (TEM): 50 nm.

A film thickness of ∼150 nm for the single-layer mesoporous silica films was determined *via* SEM. The single-layer films typically showed regular arrangements of layers along the film cross section, as deduced from the presence of fringes in the XRR spectra (Fig. 2). These fringes were differently pronounced and less pronounced for the TEOS fraction of 80 mol%. Parallel to the substrate surface, there were ordered domains, as deduced from the GISAXS results showing clear patterns (Fig. 2). The domain orientations decreased gradually with increasing TEOS fraction from 70 mol% to 100 mol% (Fig. 2c). Upon reducing the TEOS fraction to 50 mol%, the domains were neither oriented nor ordered, and thus, no patterns or halos are visible in the GISAXS images (Fig. 2o). Due to mesopores with diameters of ∼6–7 nm, as indicated by the TEM images, the films inherited pore volume fractions of 50 vol%, as determined *via* ellipsometry and the Bruggeman effective medium approximation^34^ (Table S1 and Fig. S1†).

**Fig. 3:**
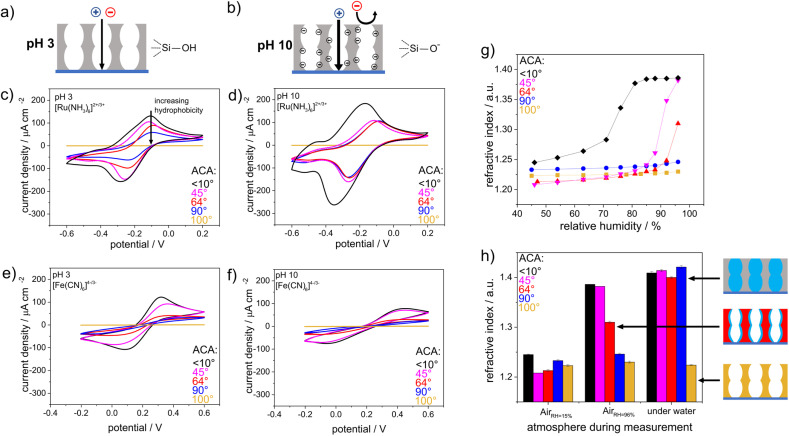
Schematic view of an uncharged silica film at acidic solution pH (a) and a negatively charged silica film at basic solution pH (b) with indicated permselectivity of a positively and negatively charged probe molecule. CV measurements of mesoporous silica films with differing wettabilities at solution pH 3 (c) and pH 10 (d) using the positively charged probe molecule [Ru(NH_3_)_6_]^2+/3+^. CV measurements of mesoporous silica films with differing wettabilities at solution pH 3 (e) and pH 10 (f) using the negatively charged probe molecule [Fe(CN)_6_]^4−/3−^. (g) Influence of the wetting properties on the adsorption of water, as reflected by the refractive index dependence on the relative humidity. (h) Refractive index determined by ellipsometry for silica films with differing wetting properties. The measurements were performed at 15% RH, 96% RH and under water.

**Fig. 4:**
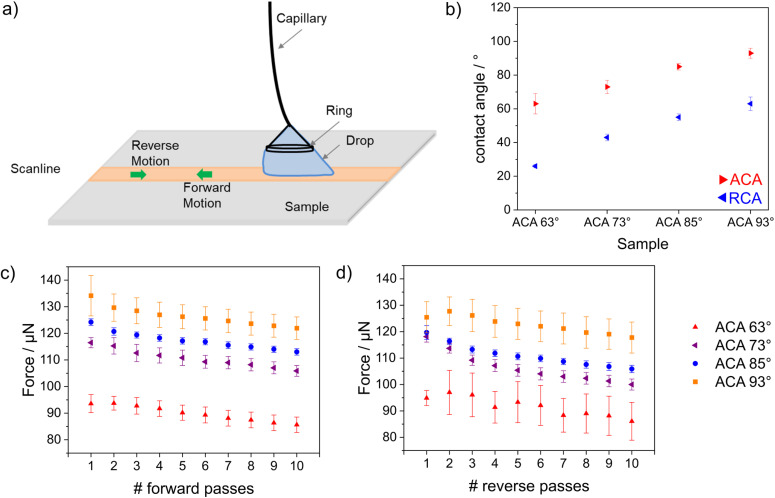
(a) A schematic of the method used for measuring drop friction forces. (b) Dynamic advancing and receding contact angles during the forward motion of pass 2. (c) Average kinetic friction force during forward motion for the samples with different wettabilities. (d) Average kinetic friction force during reverse motion for the samples with different wettabilities. The observed frictional force trend for different samples was consistent for both motions. The error bars represent the statistical variations of force in the kinetic region within a scanline.

The wettability- and pH-dependent molecular transport into these single-layer mesoporous films were investigated with CV. Due to silanol groups at the mesopore wall that can either be protonated or deprotonated, a change in the solution pH from acidic to basic led to an increase in the negative charge density at the mesopore walls.^35–37^ These deprotonated and thus negatively charged silanolate groups lead to electrostatic attractions with the mesopore wall and the cationic probe molecules, while anionic probe molecules were electrostatically excluded. As the mesopore size was within the range of the Debye screening length,^38^ this electrostatic repulsion led to exclusion of identically charged molecules from the mesopores, which was reflected by the absence of a peak current density in the cyclic voltammogram (Fig. 3b). For acidic pHs and thus neutrally charged mesopore walls, similar ionic mesopore accessibility for the positively and negatively charged molecules is expected (Fig. 3a). Fig. 3c and e show mesopore accessibility for the probe molecules [Fe(CN)_6_]^4−/3−^ and [Ru(NH_3_)_6_]^2+/3+^ at pH 3 and thus for neutrally charged mesopore walls. A direct influence of wettability on mass transport was detected. An increasing exclusion with increasing hydrophobicity of the mesoporous silica film was indicated by decreasing peak current densities with increasing hydrophobicity. For an ACA of 100°, no mesopore accessibility was detectable, which was reflected by the absence of a peak current in the cyclic voltammogram (Fig. 3c and e orange). It should be noted that the degree of order and orientation of mesopores within the films (Fig. 2) are known to also influence mass transport of the redox probe.^39,40^ Here, the silica film with a molar TEOS fraction of 100 mol% showed the highest peak current density (Fig. 3c, black), while it consisted of unoriented domains in one zone but ordered domains along the film thickness (Fig. 2c). In contrast, the silica film with a TEOS fraction of 50 mol% showed an unordered and unoriented mesoporous structure (Fig. 2o), but there was no trend in orientation and order with decreasing TEOS fraction from 100 mol% to 50 mol%. While all films showed relatively high porosity according to the ellipsometry data, the main effect on molecular transport control resulted from the increasing hydrophobicity of the film and cannot mainly be ascribed to a structural influence. Upon changing the solution pH from acidic to basic and thus generating negatively charged mesopore walls, the peak current densities in the cyclic voltammograms for mesoporous films with ACAs of 45° and 90° were comparable, showing [Ru(NH_3_)_6_]^2+/3+^ diffusion into the mesoporous film at basic pH (Fig. 3d and f). Interestingly, the voltammograms were similar to those for [Ru(NH_3_)_6_]^2+/3+^ diffusion at acidic pHs (Fig. 3c) and showed less peak broadening as well as lower peak current densities than those for pure mesoporous silica films (Fig. 3d black). This indicated a reduced influence of the remaining silanol groups at the mesopore walls after methylsilane cocondensation at basic pHs for ACAs between 45° and 90°. Finally, regardless of the probe molecule and the solution pH, no molecular transport was observed for mesoporous films with ACAs above 90° and 100°, resulting in a threshold ACA of ∼90°. This was supported by ellipsometry measurements performed in contact with fluid water, which showed water filled mesopores for ACAs below 90° as a consequence of water imbibition into the mesoporous silica film but fluid water exclusion for mesoporous films with ACAs higher than 90° (Fig. 3g and h). For visualization, a positively charged fluorophore, ATTO647N, was applied to the mesoporous films. No accumulation of ATTO647N was observed inside the mesoporous silica films with ACAs above 90° (Fig. S2†), which was consistent with the ellipsometry and CV results. To understand drop fluid transport, the friction force of water drops ^29,41,42^ was measured on a mesoporous surface with adjustable wettability (Fig. 4a). The wettability was gradually adjusted *via* post-functionalization of a mesoporous silica film while ensuring an identical mesoporous structure.

After post-grafting with a perfluorinated silane, both contact angles, the ACA and the receding contact angle (RCA) increased with increasing hydrophobicity. The samples with ACAs of 93°, 85° and 73° showed similar hysteresis (*θ*_a_ − *θ*_r_) ≈ 30°, and the sample with an ACA of 63° showed slightly higher hysteresis ≈ 37° (Fig. 4b). The lowest kinetic friction was measured for the sample with an ACA of 63°. The friction force was constant in both the forward and reverse motions (pass 1 in Fig. 4c and d). The average kinetic friction decreased with increasing numbers of forward and reverse passes (Fig. 4c and d). This decrease was consistent among all the samples, irrespective of the presence of an imbibition effect on the surface. The decrease in force for each sample followed the same slope. Thus, the decrease in force with each pass was attributed to evaporation of the drop. For all passes, the maximum friction was observed for the sample with an ACA of 93°, followed by the samples with ACAs of 85°, 73°, and 63°. This trend indicated a self-lubricating effect, which increased with decreasing contact angle. During drop contact with the surface, water was imbibed into the silica film, leading to a reduced frictional force between the silica film and the droplet. The DoFFI measurements indicated that imbibition occurred at a rate higher than the drop sliding speed (4 mm s^−1^), which was consistent with previous work by our group.^32,43^ For all passes during forward motion, we measured average forces in the range of 130–110 μN for the sample with an ACA of 93°, 120–105 μN for the sample with an ACA of 85°, 120–100 μN for the sample with an ACA of 73°, and 105–80 μN for the sample with an ACA of 63°. For all passes during reverse motion, average forces in the range of 140–120 μN for the sample with an ACA of 93°, 125–110 μN for the sample with an ACA of 85°, 120–105 μN for the sample with an ACA of 73°, and 95–80 μN for the sample with an ACA of 63° were measured. The observed force ranges matched quite well for the forward and reverse passes on all the samples. Any observed difference in the force magnitude between forward and reverse motion was attributed to eccentricity arising out of ring attachment.

**Fig. 5:**
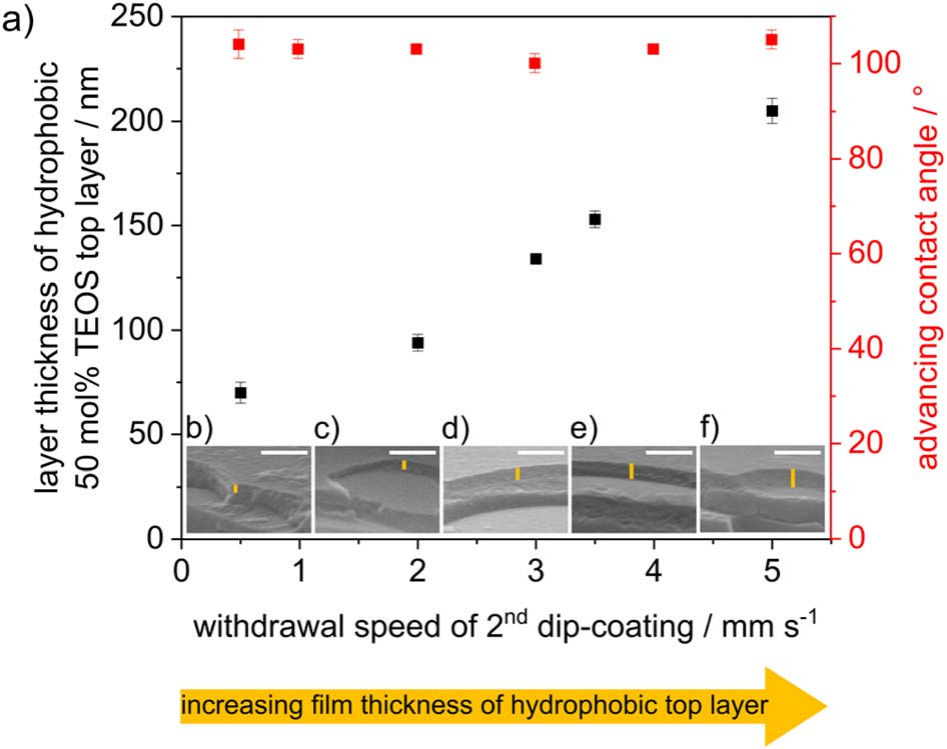
(a) Thickness of the top layer (black) determined by ellipsometry and advancing water contact angle (red) of mesoporous silica film consisting of a hydrophilic bottom layer and a hydrophobic top layer and dependence of the withdrawal speed, while dip-coating of the second layer. (b–f) SEM images of double layer silica film using different withdrawal speeds of dip-coating of the second layer. Scale bar (SEM): 500 nm.

Cocondensation results in wettability and pH-dependent transport characteristics similar to post-grafting in single-layer mesoporous films.^43^ DoFFI shows homogeneous surfaces and decreasing friction force with decreasing ACA. To investigate the influence of nanoscopic wetting asymmetry on molecular transport, condensation, and fluid distribution, we assembled these individual layers into defined double layer step gradients. First, double-layer mesoporous silica films consisting of a hydrophilic bottom layer (100 mol% TEOS, ACA < 10°) and a hydrophobic top layer (50 mol% TEOS, ACÃ 100°) were fabricated *via* consecutive dip-coating steps, in accordance with Stanzel *et al.*^44^ The withdrawal speed of the first dip-coating was kept constant between all samples, resulting in a layer thickness of ∼175 nm for the bottom mesoporous silica layer, as determined from ellipsometry and SEM measurements (Fig. 5b–f and Table S2†). The thickness of the mesoporous top layer was varied by changing the withdrawal speed of the second dip-coating step. With increasing withdrawal speed, the layer thickness was gradually adjusted from 70 nm to 205 nm, as determined by ellipsometry and SEM (Fig. 5), in agreement with the literature.^45^ The porosities of the silica films were determined to be in the range of ∼40 vol% for both layers, as analyzed by ellipsometry using the Bruggeman effective medium approximation^34^ (Table S3†). The ACA of the double layer film surface remained at ∼100° independent of the film thickness of the top hydrophobic layer (Fig. 5).

**Fig. 6:**
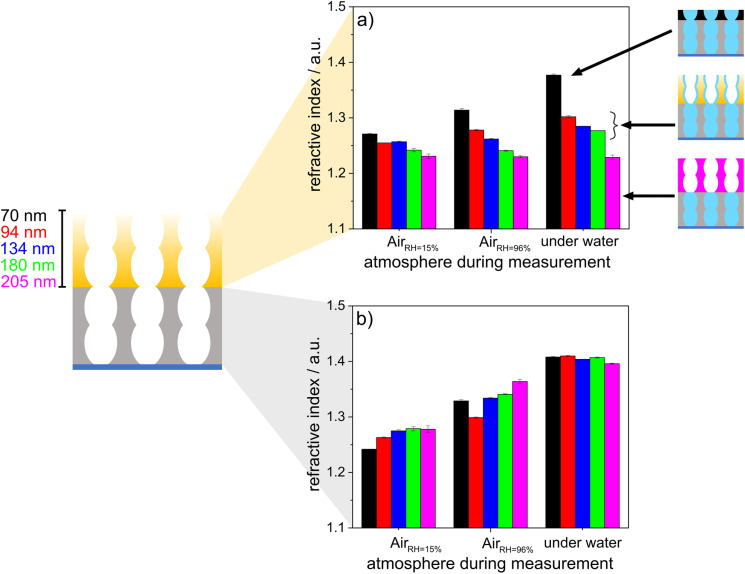
Refractive index of a hydrophobic top layer (a) and a hydrophilic bottom layer (b) determined by ellipsometry for double layer silica films. The thickness of the bottom layer was held constant, whereas the thickness of the top layer was gradually increased from 70 nm to 205 nm. The measurements were performed at 15% RH, 96% RH and under water.

To investigate the effects of wetting asymmetry on water condensation, water adsorption and water distribution in double-layer silica films, ellipsometry measurements were performed under varying atmospheric conditions. Fig. 6 shows the refractive indices of the hydrophobic top layer (Fig. 6a) and hydrophilic bottom layer (Fig. 6b) in contact with dry air (RH = 15%), wet air (RH = 96%) and fluid water. Water-filled mesopores are expected to show a refractive index of ∼1.4, as estimated from the effective medium theory^34^ with the refractive index of water (*n* = 1.33),^46^ a refractive index for nonporous silica of *n* = 1.455 (ref. 47) and the mesopore volume fractions from measurements in dry conditions. When the relative humidity was increased from 15% to 96%, the refractive index of the hydrophobic top layer within the mesoporous double-layer film did not increase significantly except for the thinnest top layer of 70 nm. Interestingly, this refractive index increase was not observed for the hydrophobic single-layer silica film. No water condensation into the hydrophobic single-layer film was observed at 96% relative humidity or in contact with fluid water (Fig. 3).

When the mesoporous double layer silica films were immersed in fluid water at pH > 7, the hydrophobic top layer seemed to be partly filled with water, probably due to adsorption at the remaining negatively charged silanol groups on the pore walls. When the layer thickness of the hydrophobic top layer was increased, the influence of the water-filled hydrophilic bottom layer on the water adsorption in the hydrophobic top layer decreased gradually with increasing layer thickness, showing water adsorption and a slight refractive index increase in the top layer. For top layer thicknesses up to 205 nm, the refractive index of the top layer did not increase upon contact with liquid water, while the refractive index of 1.4 for the hydrophilic bottom layer still indicated water-filled mesopores, probably due to condensation. Consequently, this indicated that water vapor passed the hydrophobic top layer, which was in contact with fluid water regardless of the hydrophobic top layer thickness. Then, water condensed in the hydrophilic bottom layer and filled the bottom layer while the top layer remained dry. Water imbibition or condensation inside the silica bottom layer was also indicated by interferometry (Fig. S3†). Together with the decreasing drop friction forces with decreasing ACA, this gives a tool, with which to design drop friction and lubrication of mesoporous multilayer films with the relative humidity.

**Fig. 7:**
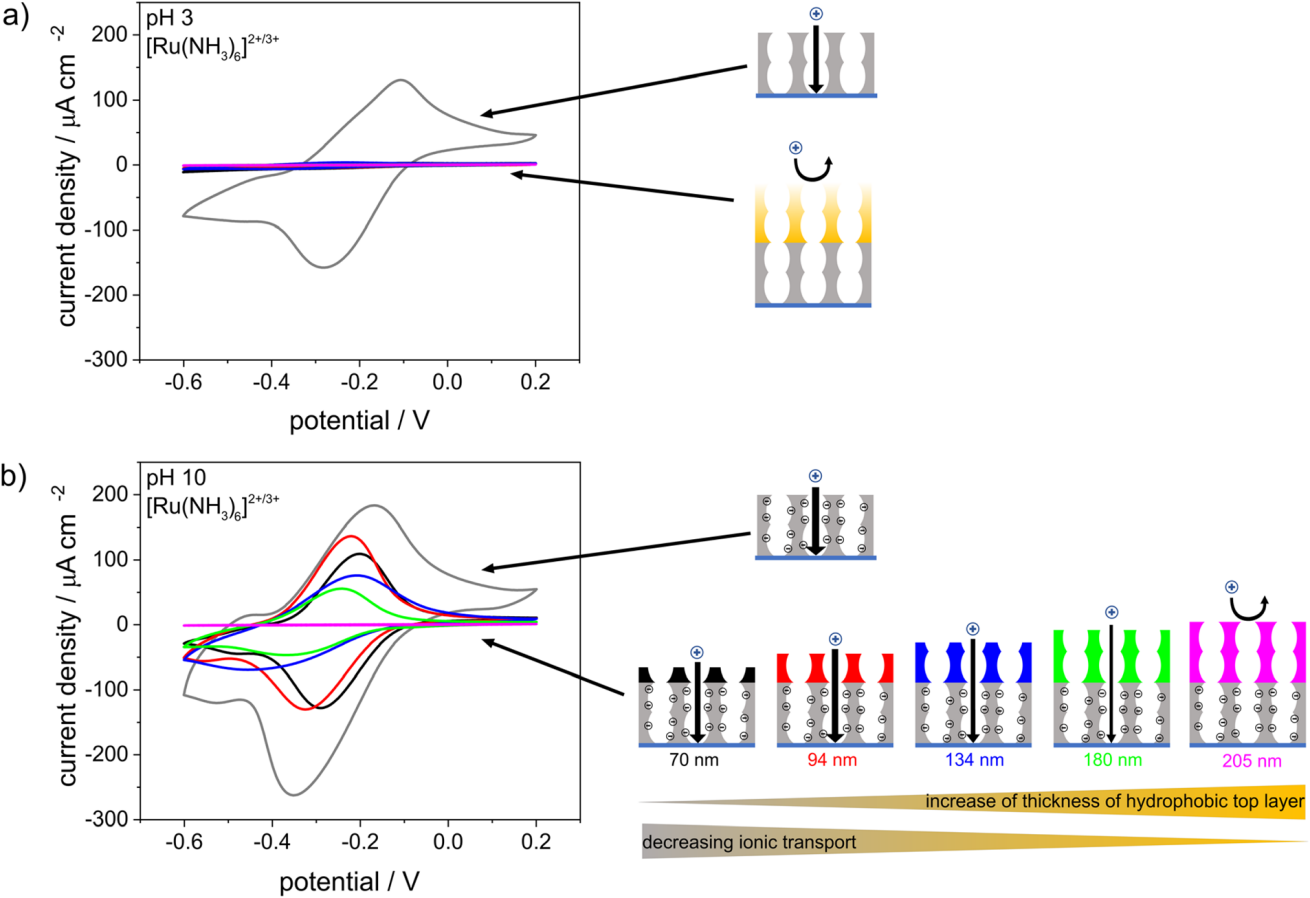
Cyclic voltammograms of a single layer mesoporous silica film (gray) and of double layer silica films with hydrophilic bottom layers and a hydrophobic top layers. The thickness of the bottom layer was held constant, whereas the thicknesses of the top layers were gradually increased from 70 nm to 205 nm. Measurements were performed at acidic (a) and basic (b) pH values using 10^−3^m [Ru(NH_3_)_6_]^2+/3+^ as a probe molecule in 0.1 m aqueous KCl solution. The scan rate was 100 mV s^−1^.

CV was used to investigate the influence of the pH-responsive, hydrophilic bottom layer on the ionic mesopore accessibility of the positively charged probe molecule [Ru(NH_3_)_6_]^2+/3+^ as a function of the solution pH (Fig. 7). At an acidic solution pH of 3 and thus in the absence of negatively charged silanol groups at the mesopore wall, water and thus the dissolved ions were excluded from the mesoporous film regardless of the thickness of the hydrophobic top layer (Fig. 7a). At basic solution pHs and thus in the presence of negatively charged mesopore walls,^35–37^ the mesoporous films were accessible to [Ru(NH_3_)_6_]^2+/3+^ even with the thickest top layer of 205 nm and an ACA of ∼100°. However, the peak current density decreased with increasing hydrophobic top layer thickness until total exclusion of [Ru(NH_3_)_6_]^2+/3+^ was observed for hydrophobic mesoporous top layer thicknesses of 180–205 nm. In comparison, a single layer hydrophobic silica film with a film thickness of ∼150 nm showed no mesopore accessibility for a positively charged probe molecule at basic solution pHs (Fig. 3). Therefore, it can be concluded that the change in mesopore accessibility with varying hydrophobic top layer thickness was affected by the hydrophilic bottom layer and its pH-responsive mesopore wall charges. The influence of the bottom layer on water sorption and molecular transport of the top layer in double-layer silica films with different pore sizes or surface charges was shown by Fuertes *et al.*^24^ and Stanzel *et al.*^44^ Interestingly, the shapes of the cyclic voltammograms for the double layer silica films at basic solution pHs indicated transport along the mesopore wall and strong electrostatic attraction, as shown by Rohlfing *et al.* for mesoporous silica films with redox active probe molecules covalently attached to the inner pore walls.^48^ The peak-to-peak separations shifted from 175 V for a hydrophilic single-layer silica film (Fig. 7b, gray) to 96 mV, 101 mV, and 122 mV for double-layer silica films with 70 nm-, 94 nm- and 180 nm-thick hydrophobic top layers, respectively. Since the ellipsometry measurements made in contact with fluid water (Fig. 6a) showed minimal water adsorption inside the hydrophobic top layer, ionic transport probably occurred through the minimal amount of water adsorbed to the pore wall, as previously proposed for single-layer silica films.^43^ In addition, mass transport through the hydrophobic top layer may have been increased by electroconvection due to the potentials applied during the CV measurements. Experimental studies showed that voltage-driven convective flow occurred in dilute electrolyte solutions due to an applied electric field force, and this increased mass transfer.^49–51^ Interestingly, after CV measurements performed at basic pHs, the positively charged probe molecule [Ru(NH_3_)_6_]^2+/3+^ could not be removed from the double-layer silica film by extraction with basic or acidic water, resulting in an increased peak current density when the CV measurements were repeated at acidic solution pHs (Fig. S4†). Trapping of [Ru(NH_3_)_6_]^2+/3+^ was also observed with the fluorophore methylene blue used as a probe molecule and fluorescence image acquisition as the detection method (Fig. S5†). After the CV measurements and application of a potential at a solution pH of 10, the fluorophore was fully removed from the hydrophilic single-layer silica film after extraction with acidic water. Until the thickness of the hydrophobic top layer reached 180 nm, residues of the fluorophore remained inside the film even after extraction. Further increases in the thickness of the hydrophobic top layer film caused exclusion of the fluorophore, as was also shown for the positively charged probe molecule in the CV measurements (Fig. 7). Fluorescence microscopy experiments with a positively charged fluorophore at basic pH and without applying any charge showed very slight fluorophore adsorption inside the mesoporous silica films with top layer thicknesses up to 134 nm, while thicker top layers prevented fluorophore adsorption, and no significant fluorophore adsorption inside the mesoporous silica film was observed (Fig. S6†). For the ellipsometry measurements made in contact with fluid water (Fig. 6), minimal water adsorption inside the hydrophobic top layer meant only a small amount of the fluorophore diffused into the mesopores without an applied voltage. This further suggested that an interplay between the charge in the lower film layer and the applied potential increased transport.

## Experimental section

### Materials

Pluronic® F127, DEDMS (97%), TEMS (99%) and Emplura® ethanol were purchased from Sigma Aldrich. TEOS (98%) was received from Alfar Aesar. All chemicals were used as received unless noted otherwise.

### Preparation of mesoporous silica thin films

In the syntheses of mesoporous silica thin films with adjustable wetting properties, sol–gel solutions with the following molar ratios were used: *x* TEOS : 1 − *x* (0.6 TEMS : 0.4 DEDMS) : 0.0075 F127 : 10H_2_O : 40EtOH : 0.03 HCl (37%). The solution was stirred overnight before dip-coating to produce single-layer thin films with a withdrawal speed of 2 mm s^−1^ at a relative humidity (RH) of ∼60% and a temperature of ∼23 °C. Freshly deposited mesoporous films were kept under these climate conditions for at least 1 h before they were thermally treated with the following oven program: 1 h at 60 °C and 1 h at 130 °C followed by heating to 350 °C with a heating rate of 1 °C min^−1^ and stabilization at 350 °C for 2 h.

The mesoporous double layer silica films were prepared according to the method of Stanzel *et al.*^44^ For fabrication of the double-layer silica films, the first layer was dip-coated with a withdrawal speed of 2 mm s^−1^, kept at ambient conditions for 1 h and stabilized by heating at 60 °C and 130 °C for 1 h, respectively. The mesostructured bottom layer was then covered with a second silica film *via* dip coating using withdrawal speeds of 0.5, 2, 3, 4.5 or 5 mm s^−1^ and stored at 23 °C and 50% relative humidity for 1 h, followed by the oven program used for single-layer silica films of 60 °C and 130 °C for 1 h and 350 °C for 2 h.

### Vapor-phase deposition of 1*H*,1*H*,2*H*,2*H*-perfluorooctyl dimethylchlorosilane (PFODMCS)

In accordance with Khalil *et al.*,^43^ vapor phase deposition of PFODMCS was performed in a vacuum chamber with a volume of 1 L at room temperature. Initially, the mesoporous silica films (100 mol% TEOS) were cleaned with ethanol and dried with pressurized nitrogen before being placed in the reaction chamber under a nitrogen counterflow. The chamber containing the samples was alternatingly evacuated and flushed with gaseous nitrogen three times before 10 μL of PFODMCS was placed onto the bottom of the chamber under nitrogen counterflow. Afterward, a reduced pressure of 100 mbar was applied to the chamber and maintained for a fixed duration. Finally, the samples were rinsed and extracted with toluene and ethanol before being stored under ambient conditions.

### Contact angle

Macroscopic contact angles (CAs) were measured with an OCA 35 device from DataPhysics Instruments using SCA 4.5.2 software and the sessile drop method under a standard atmosphere (*T* = 23 °C, RH = 60%). Drop volumes of 2 μL were used, and the CAs were obtained by fitting the droplet shape with the approximation algorithm of the SCA software. Dynamic CAs were measured by increasing and decreasing the droplet volume from 2 μL to 10 μL with a dosing speed of 0.2 μL s^−1^. Simultaneously, a video was recorded and later analyzed to determine the dynamic CA with the SCA 4.5.2 software.

### Ellipsometry

The film thicknesses and refractive indices of the single-layer and double-layer mesoporous silica films were determined with ellipsometry using an Accurion Nanofilm EP3 ellipsometer. The measurements were carried out with angles of incidence between 38° and 68° at a step size of 2° at three measuring positions along the withdrawal direction of the dip coating. Measurements were taken in one-zone mode. A laser with a wavelength of 658 nm was used.

For the evaluation, a single-layer model was created for the mesoporous films on silicon substrates (Si wafer, SiO_*x*_ layer, mesoporous SiO_2_ layer) with the program EP4-Model (version 1.2.0) from Accurion; this was used to describe the surfaces with fitting limits of 100–250 nm for film thicknesses of 100–250 nm and 1.0–1.5 for refractive indices. For the double layer silica films, the mesoporous SiO_2_ layer was split into two, while fixing the thickness of the 180 nm bottom layer and fitting two different refractive indices and the thickness of the top layer *via* iteration between 50 and 250 nm.

A humidity controller and the program Regul'Hum (version 3.3) from SolGelWay was used to keep the relative humidity at 15% for the standard ellipsometry measurements, or it was increased/decreased to monitor water sorption in the mesoporous silica film according to Boissiere *et al.*^52^ In addition, ellipsometry measurements were performed under water.

### Scanning electron microscopy (SEM)

A ZEISS DSM 962 scanning electron microscope with an SE detector was used (resolution: 10 nm lateral). The samples were cut to size and glued to a sample holder with a carbon film. For cross-sectional imaging, the samples were fixed with a conductive tape strip. The samples were covered with a 7 nm layer of Pd/Pt alloy using a Cression 208 HR Sputter Coater.

### Transmission electron microscopy (TEM)

Transmission electron micrographs were recorded by Raheleh Pardehkhorram (TU Darmstadt, Andrieu-Brunsen) with a JEOL JEM 2100F TEM operating at an accelerating voltage of 200 kV. For the examinations of the porous silica films, the samples were scraped off and suspended in ethanol. Before placing one drop on a TEM mesh, they were treated for 5–10 min in an ultrasonic bath.

### Cyclic voltammetry (CV)

The CV measurements were performed with an Autolab PGSTAT302N potentiostat from Metrohm. The anionic probe molecules [Fe(CN)_6_]^4−/3−^ and the cationic probe molecule [Ru(NH_3_)_6_]^2+/3+^ (each 1 mm in 0.1 m aqueous KCl solution) were used. The indium tin oxide layer of the respective substrates supporting the mesoporous film (Delta Technologies, resistance of 4–8 Ω) served as the working electrode. A Ag/AgCl electrode (BASi RE-6) served as the reference electrode, and a graphite electrode served as the counter electrode. The pH-dependent mesopore accessibility was investigated by adjusting the solution pH to 3 and 10 by adding hydrochloric acid and sodium hydroxide solutions and using a pH meter (Seven Compact S220 by Mettler Toledo). The measurements with [Fe(CN)_6_]^4−/3−^ were carried out with a voltage range of −0.2 V to 0.6 V, and the measurements with [Ru(NH_3_)_6_]^2+/3+^ were carried out with a voltage of −0.6 V to 0.3 V. Measurements were taken at scan rates of 200 mV s^−1^, 100 mV s^−1^, 25 mV s^−1^, 300 mV s^−1^, 500 mV s^−1^ and finally again at 200 mV s^−1^. Three cycles were run at each scan rate to ensure that equilibrium had been reached, and the data were only evaluated when the 200 mV s^−1^ scan rates showed comparable results. For the evaluation, a scan rate of 100 mV s^−1^ was used in each case. After including the electrode surface area (0.21 cm^−1^), the current density *J* in μA cm^−2^ was plotted against the potential *E* in V.

### Drop friction force microscopy (DoFFI)

A glass capillary of dimension 0.05 mm × 0.5 mm × 50 mm is attached to a metal holder *via* an epoxy resin (UHU plus 2 Component Epoxy). The spring constant of the capillary is then determined *via* vibrational method. The capillary is given a gentle blow and the subsequent time scale of vibration is noted with the help of CMOS camera (Krüss DSA 100). With the help of eqn (1), we estimate the spring constant to be in the order of 100 μN mm^−1^. A Milli-Q water (Sartorius AG.) drop of 15 μL is immobilized on a surface with a help of this capillary. The end of the glass sensor is equipped with a centred metal ring of diameter ≈ 2 mm. The ring is made out of metal wire having 0.5 mm diameter. The ring serves the purpose of holding and sliding the drop even on hydrophilic surfaces. The stage onto which the sample is placed is then moved at 4 mm s^−1^ speed, which results in deflection of the glass capillary sensor. The deflection data is acquired at the speed of 30 fps using CMOS camera. This deflection of the senor is quantified by the image analysis using in-house MATLAB script. The data obtained is then multiplied with the spring constant to obtain friction force. For a sample, we perform measurements along two scanlines. On each scan line, the drop is displaced 25 mm. Each scanline comprises of 10 forward passes and 10 reverse motions.1
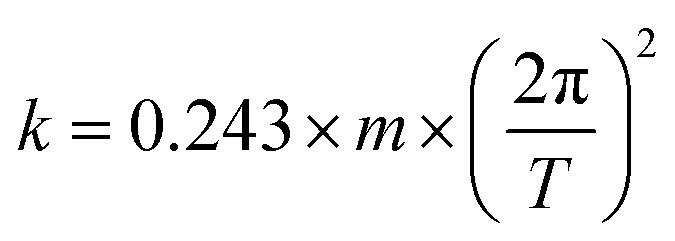


### Fluorescence microscopy

A glass capillary with dimensions of 0.05 mm × 0.5 mm × 50 mm was attached to a metal holder with an epoxy resin (UHU plus 2 Component Epoxy). The spring constant of the capillary was then determined *via* the vibrational method. The capillary was given a gentle blow, and the subsequent time scale of the vibrations was noted with the help of a CMOS camera (Krüss DSA 100). We estimated the spring constant to be on the order of 100 μN mm^−1^. A 15 μL Milli-Q water (Sartorius AG.) drop was immobilized on a surface with the help of this capillary. The end of the glass sensor was equipped with a centered metal ring with a diameter of ∼2 mm. The ring was made of metal wire with a 0.5 mm diameter. The ring served the purpose of holding and sliding the drop even on the hydrophilic surfaces. The stage onto which the sample was placed was then moved at a speed of 4 mm s^−1^, which resulted in deflection of the glass capillary sensor. The deflection data were acquired at a speed of 30 fps with a CMOS camera. This deflection of the sensor was quantified by image analysis with an in-house MATLAB script. The data obtained were then multiplied by the spring constant to obtain the frictional force. For each sample, we performed measurements along two scanlines. On each scan line, the drop was displaced by 25 mm. Each scanline comprised 10 forward passes and 10 reverse motions.

### Imager

Fluorescence image acquisition was performed with a Vilber Fusion FX system (VILBER LOURMAT Deutschland GmbH, Eberhardzell, Germany). For detection of methylene blue, an excitation filter with a band between 620 and 660 nm (C640) and an emission filter with a band between 660 and 730 nm (F-695) were used. The excitation time was 7 s. Prior to imaging, the samples were stained for ∼7 min in a methylene blue solution (1 mm in 0.1 m aqueous KCl solution) while applying a potential using the cyclic voltammetry setup with the same procedure. The measurements were carried out at over a voltage range of −0.6 V to 0.3 V. Measurements were taken at scan rates of 200 mV s^−1^, 100 mV s^−1^, 25 mV s^−1^, 300 mV s^−1^, 500 mV s^−1^ and finally again at 200 mV s^−1^. Three cycles were run at each scan rate to ensure that equilibrium had been reached. For the evaluations, a scan rate of 100 mV s^−1^ was considered in each case. The procedure was followed by washing for 10 min in a 0.01 M aqueous HCl solution.

### X-ray reflectometry (XRR) and grazing-incidence small-angle X-ray scattering (GISAXS)

XRR and GISAXS experiments were performed with a XEUSS 1.0 SAXS setup (XENOCs, Grenoble, France) at a temperature of 25 °C and an RH less than 20%. Monochromatic X-rays (*λ* = 0.15419 nm) were produced with a GENIX 3D microfocus tube. The incoming X-ray beam was collimated to give a size at the sample position of 0.15 × 0.15 mm^2^. Scattered photons were detected with a PILATUS 100 K detector placed at a sample-to-detector distance of *D* = 2500 mm (calibrated using silver behenate as a standard).

### Interferometry

To investigate whether water accumulated in the hydrophilic layer, the reflected light intensities were analyzed. In these experiments, a droplet of distilled water with a volume of 2 μL was deposited on the initially dry mesoporous silica film, which had not been treated after the coating process. Three laser beams with wavelengths of 457 nm, 532 nm and 639 nm were directed at the sample with normal incidence angles. The reflected light was measured with a color camera (iDS UI-3080SE-C-HQ). A Navitar objective with a maximum magnification of 14 was used. The measurements were obtained approximately two minutes after the droplet was deposited on the substrate. The measured intensity with the present droplet was compared to the background intensity. This was done for each color channel (red, green and blue). The experiments were performed at a relative humidity of approximately 57% and a temperature of approximately 24 °C.

## Conclusions

Mesoporous silica films with adjustable wettabilities were synthesized *via* cocondensation of TEOS, TEMS and DEDMS. The direct influence of the mesoporous layer wettability on pH-dependent mass transport was demonstrated in accordance with previous work.^43^ Molecular transport was blocked for mesoporous films with ACAs above 90°. DoFFI showed a gradually decreasing frictional force with decreasing ACA, indicating a self-lubricating effect of the mesoporous silica film, which was ascribed to faster fluid imbibition than the drop scanning rate during the DoFFI measurement.

Assembling the mesoporous silica single layers into mesoporous double layer films with wettability step gradients consisting of a hydrophilic mesoporous silica bottom layer with constant thickness and a hydrophobic mesoporous silica top layer (ACA ∼100°) with increasing layer thicknesses of up to 205 nm resulted in top layer thickness- and pH-dependent fluid and ionic mesopore accessibility. At 96% relative humidity, water condensation occurred in the hydrophilic bottom layer, while the water content in the hydrophobic top layer depended on the layer thickness. For top layer thicknesses of 180–205 nm, the refractive index of the hydrophobic top layer remained constant, indicating no water had condensed. At lower film thicknesses for the hydrophobic mesoporous top layer, water seemed to adsorb in the top layer when the bottom layer contained condensed water (contact with 96% relative humidity). In contact with fluid water (pH > 7), similar behavior was observed with partly filled mesopores in the hydrophobic top layer, while the hydrophilic mesoporous bottom layer was filled, probably due to water condensation.

The ionic mesopore accessibility also decreased with increasing film thickness of the hydrophobic top layer. Full exclusion of the positively charged probe molecule, even at a basic solution pH, and thus under electrostatic attraction of the probe molecule, was observed for hydrophobic mesoporous top layer thicknesses of 180–205 nm. Below a hydrophobic top layer thickness of 180 nm, the adsorbed water layer in the hydrophobic mesoporous top layer enabled ion transport *via* electrostatic attraction and thus for basic pH values. Interestingly, this process led to irreversible trapping and loading of the ions inside the mesopores, especially for the hydrophilic bottom layer. The present approach of gradual mesoporous wettability design together with wettability step gradient formation showed the dependence of nanoscale asymmetric wettability architecture on water condensation, as well as on ionic mesopore accessibility. It should be noted that the mesoporous layer was substrate supported, and the investigated layer thicknesses were limited to 70–205 nm. Nevertheless, this study provides insights into nanoscale wettability and architecture-dependent water condensation, water imbibition and the accessibility of ionic pores. Thus, the results of this study are of fundamental interest, *e.g.*, in the context of catalysis, separations, or surfaces and with new concepts for water generation from humid air.

## Conflicts of interest

There are no conflicts to declare.

## Supplementary Material

NA-005-D3NA00594A-s001
