# Supplementary material for: Molecular transport and water condensation inside mesopores with wettability step gradients
Source: Nanoscale Adv. 2023 Oct 3;5(22):6123–34. doi: 10.1039/d3na00594a (PMC10628989; doi:10.1039/d3na00594a)
Supplement: NA-005-D3NA00594A-s001 [file NA-005-D3NA00594A-s001.pdf]

Electronic Supplementary Material (ESI) for Nanoscale Advances.
This journal is © The Royal Society of Chemistry 2023

Supplementary Information

Molecular Transport and Water Condensation inside Mesopores with Wettability Step Gradients

Laura Despot[a], Chirag Hinduja[b], Robert Lehn[a], Joanna Mikolei[a], Timo Richter[c], Kilian Köbschall[c], Mathias Stanzel[a], Rüdiger Berger[b], Jeanette Hussong[c], Marcelo Ceolín[d] and Annette Andrieu-Brunsen*[a]

Mesoporous film structure by ellipsometry and SEM.

Thickness and refractive index of mesoporous silica films were determined by ellipsometry. The porosity was calculated using the Bruggeman effective medium theory.(1)

Table S1: Ellipsometry measurements (RH=15%) showing the film thickness and refractive indices of x TEOS mesoporous silica films using fitting limits of 130-230 nm for film thickness and 1.0-1.5 for refractive index. In addition, the porosity calculated via the Bruggeman effective medium theory is listed. Data presented as mean values of three fitted film thickness/refractive index ± error calculated for the respective parameter by EP4-Model (version 1.2.0) from Accurion.

TEOS fraction / mol%	film thickness / nm	refractive index	RMSE	porosity / vol%
100	166±8	1.210±0.006	<1.000	53±2
80	180±6	1.207±0.016	1.299-2.595	53±3
70	172±9	1.263±0.012	0.334-0.437	42±3
60	160±2	1.239±0.004	0.122-0.184	47±1
50	181±4	1.219±0.008	0.104-0.206	51±2

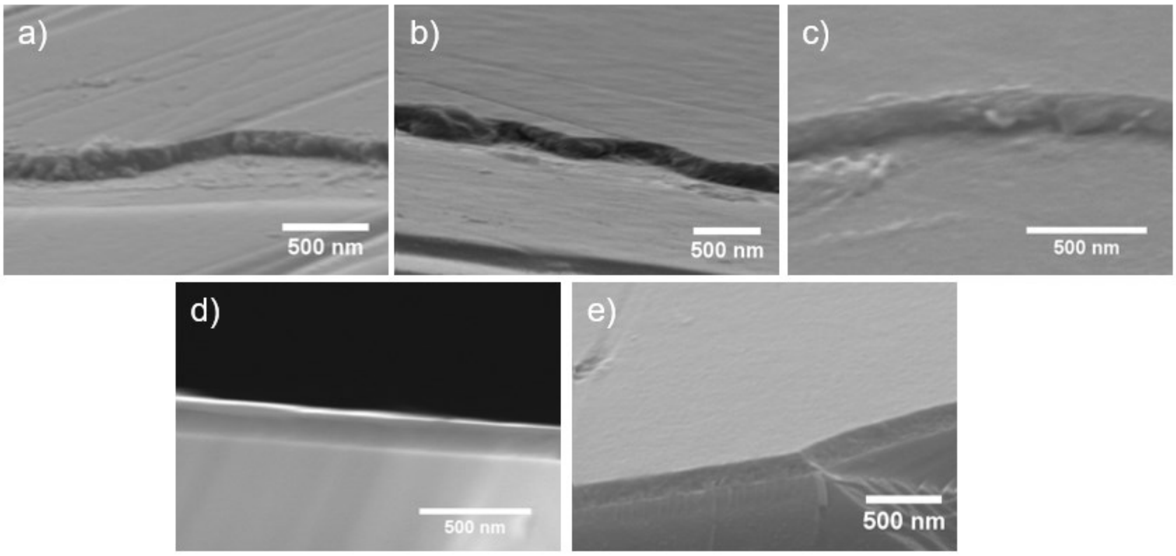

Figure S1: SEM images of x TEOS silica films with a) 100 mol% TEOS b) 80 mol% TEOS c) 70 mol% TEOS d) 60 mol% TEOS e) 50 mol% TEOS.

Layer thickness and refractive index of mesoporous double layer silica films were determined using ellipsometry (Table S2, Table S3).

Table S2: Ellipsometry measurements (RH=15%) showing the layer thickness and refractive indices of the hydrophilic bottom layer using fitting limits of 130-230 nm for film thickness and 1.0-1.5 for refractive index. In addition, the porosity calculated via the Bruggeman effective medium theory is listed. Data presented as fitted film thickness/refractive index ± error calculated for the respective parameter by EP4-Model (version 1.2.0) from Accurion.

**Withdrawal speed of 2nd dip-coating /mm s^{-1}**	**layer thickness of bottom layer / nm**	**refractive index of bottom layer**	**RMSE**	**porosity of bottom layer / vol%**
0.5	175±1	1.298±0.008	0.818	34±2
2.0	179±1	1.278±0.004	0.115	38±1
3.0	181±1	1.269±0.003	0.169	40±1
4.5	172±1	1.293±0.006	0.424	35±2
5.0	169±1	1.246±0.011	0.850	45±3

Table S3: Ellipsometry measurements (RH=15%) showing the layer thickness and the refractive indices of the hydrophobic top layer using fitting limits of 130-230 nm for film thickness and 1.0-1.5 for refractive index. In addition, the porosity calculated via the Bruggeman effective medium theory is listed. Data presented as fitted refractive index ± error calculated for the parameter by EP4-Model (version 1.2.0) from Accurion.

Withdrawal speed of 2nd dip-coating /mm s^{-1}	layer thickness of top layer / nm	refractive index of top layer	RMSE	porosity of top layer / vol%
0.5	70±5	1.189±0.010	0.818	57±3
2.0	94±4	1.280±0.001	0.115	38±1
3.0	134±1	1.278±0.002	0.169	38±1
3.5	153±4	1.265±0.005	0.424	41±1
5.0	205±6	1.225±0.007	0.850	50±2

Optimization of mesoporous film formation with gradually adjusted wettability

The sol-gel-process and mesoporous silica film formation is highly dependent on different parameters e.g. pH-value, solvent or temperature. By changing the alkoxysilane precursor from trimethoxymethylsilane (TMMS) and dimethoxydimethylsilane (DMDMS) to TEMS and DEDMS a more constant porosity (refractive index) has been achieved (Table S1). The reaction rate of hydrolysis and condensation depends on the alkyl-group and decreases with increasing steric demand.(2, 3) By choosing alkoxysilanes with ethoxy-groups as in TEOS the reaction rates are similar and the formation of the silane network is more controlled.

Fluorophore adsorption into single layer silica films with adjustable wettability

To evaluate the accessibility of mesoporous silica films with varying TEOS fractions and thus gradually varying ACA for positively charged probe molecules in the absence of an electrode and applied potential below the mesoporous film, the adsorption of the positively charged fluorophore ATTO647N(-amine) was investigated. A total internal reflection fluorescence microscope (TIRF) setup was used to only follow the fluorescence in the bottom ~190 nm of the mesoporous film. The fluorescence intensity was normalized to the background intensity. Under both, acidic (Figure S2a) and basic conditions (Figure S2b, blue and orange), films with an ACA > 90° (60 and 50 mol% TEOS) showed almost no adsorption of ATTO647N, while films with a lower ACA showed clear ATTO647N adsorption. The mesoporous films with ACA < 90° clearly show accessibility for ATTO647N. This is in accordance to ellipsometry and CV results.

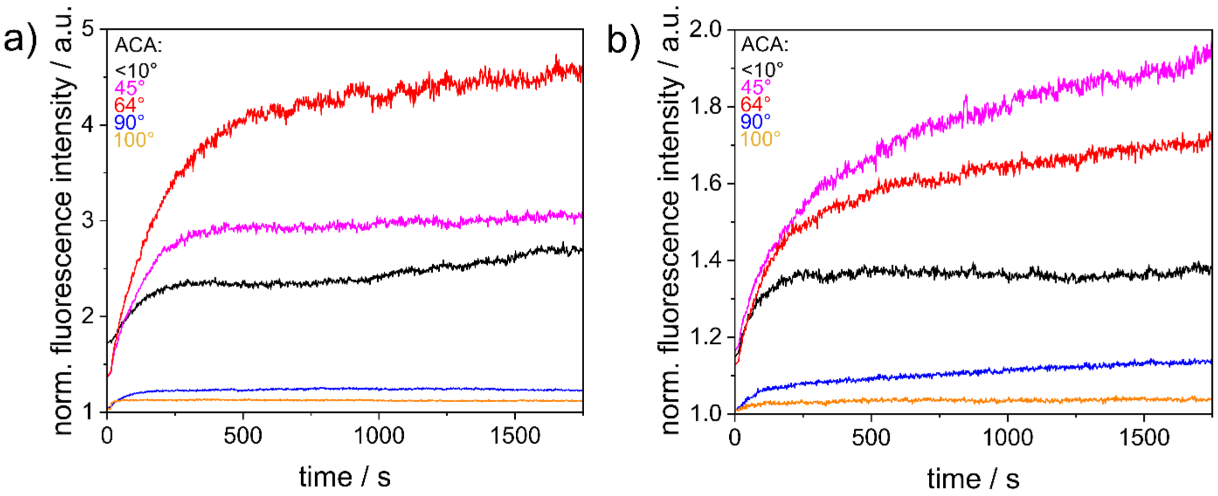

Figure S2: Average fluorescence intensity in the central illuminated region resulting from adsorption of ATTO647N from a 1 µg ml^{-1} aqueous solution at pH 2 (a) and 10 (b) into mesoporous silica films with varying ACA (<10° - 100°) over time. Intensity was normalized to the background intensity. Illumination was performed using the evanescent field of a totally internally reflected laser beam to only excite fluorophore in the lower ~250 nm of the film. Images were acquired with 0.5 fps for 45 min.

Interferometry for water imbibition investigation

In order to investigate the potential water condensation and imbibition a water droplet is placed on the mesoporous double layer film (ACA ~100°) and the sample is illuminated by a laser using three wavelengths of 457 nm, 532 nm, and 639 nm. According to the Fresnel equations(4), the reflectance is a function of the refractive index of the different layers. The presence of water in the hydrophilic layer changes its refractive index and thus the intensity of the reflected light. In the conducted experiments, a camera (iDS UI-3080SE-C-HQ) captures the reflected light. The relative change in intensity of light reflected by the substrate surrounding the droplet is exemplarily shown in Figure S3. The change in intensity around the droplet three phase contact line suggests the presence of water in the mesoporous layer. The intensity change is less pronounced at higher hydrophobic top layer thicknesses. This is in accordance with the ellipsometry results (Figure 6), which indicate water condensation and imbibition into the hydrophilic bottom layer while a thin water film is formed in the hydrophobic top layer due to adsorption.

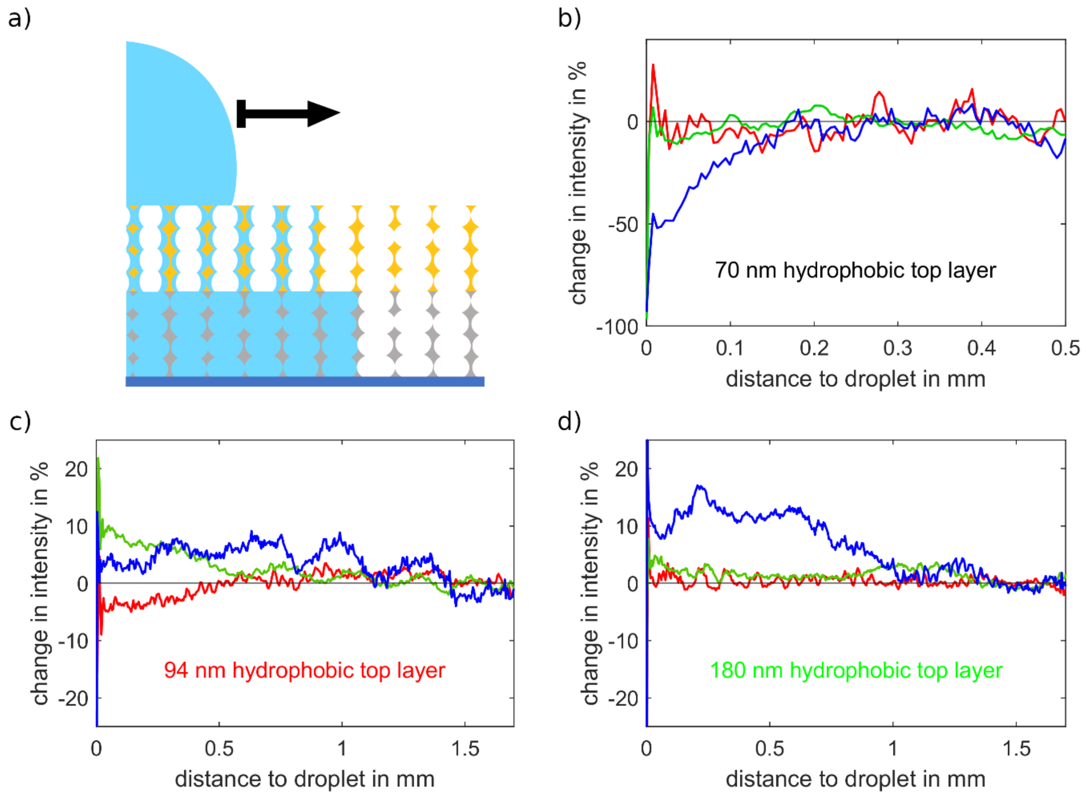

Figure S3: a) Schematic view on the region used for measurement. Relative change in reflected light intensity, which suggests the presence of water in the region close to the droplet inside he mesoporous silica double layer film with thickness of the upper, hydrophobic layer of 70 nm (a), 94 nm (b) and 180 nm (c). Measurements were performed at 56.5% RH and 24.2 °C.

Trapping of redox active probe molecule and fluorophore under a hydrophobic top layer using CV

The pH-responsive silanol groups at the mesopore pore walls can be reversibly switched between a protonated, neutrally-charged state (acidic solution pH) and a deprotonated, negatively-charged state (basic solution pH). Using a hydrophilic single layer mesoporous silica film switching to a basic solution pH leads to negatively charged pore walls and therefore electrostatic attraction between the pore wall and a cationic probe molecule. This is reflected in an increase of the peak current density in the cyclic voltammograms as compared to acidic pH (Figure S4a). After switching back to an acidic solution pH the peak current density is comparable to the one at acidic pH before basic pH was measured, which indicates a completely reversible diffusion of the probe molecule inside the pores. In contrary, when using the double layer silica film consisting of a hydrophilic, mesoporous bottom layer and a hydrophobic, mesoporous top layer with a thickness of 205 nm no peak current density is detected at acidic or at basic pH, which suggest a complete exclusion of the probe molecule regardless of the solution pH for this hydrophobic top layer thickness (Figure S4g). When reducing the hydrophobic, mesoporous top layer thickness to 70-134 nm, interestingly the counter charged probe molecule is not reversibly released when switching from basic pH back to acidic pH (Figure S4c-e). The probe molecule seems to be trapped inside the silica film due to the hydrophobic barrier. This effect increases with the layer thickness from 70 nm to 134 nm as visible by the reduced decrease in peak current density upon pH change from basic to acidic (Figure 4c-e blue, red)

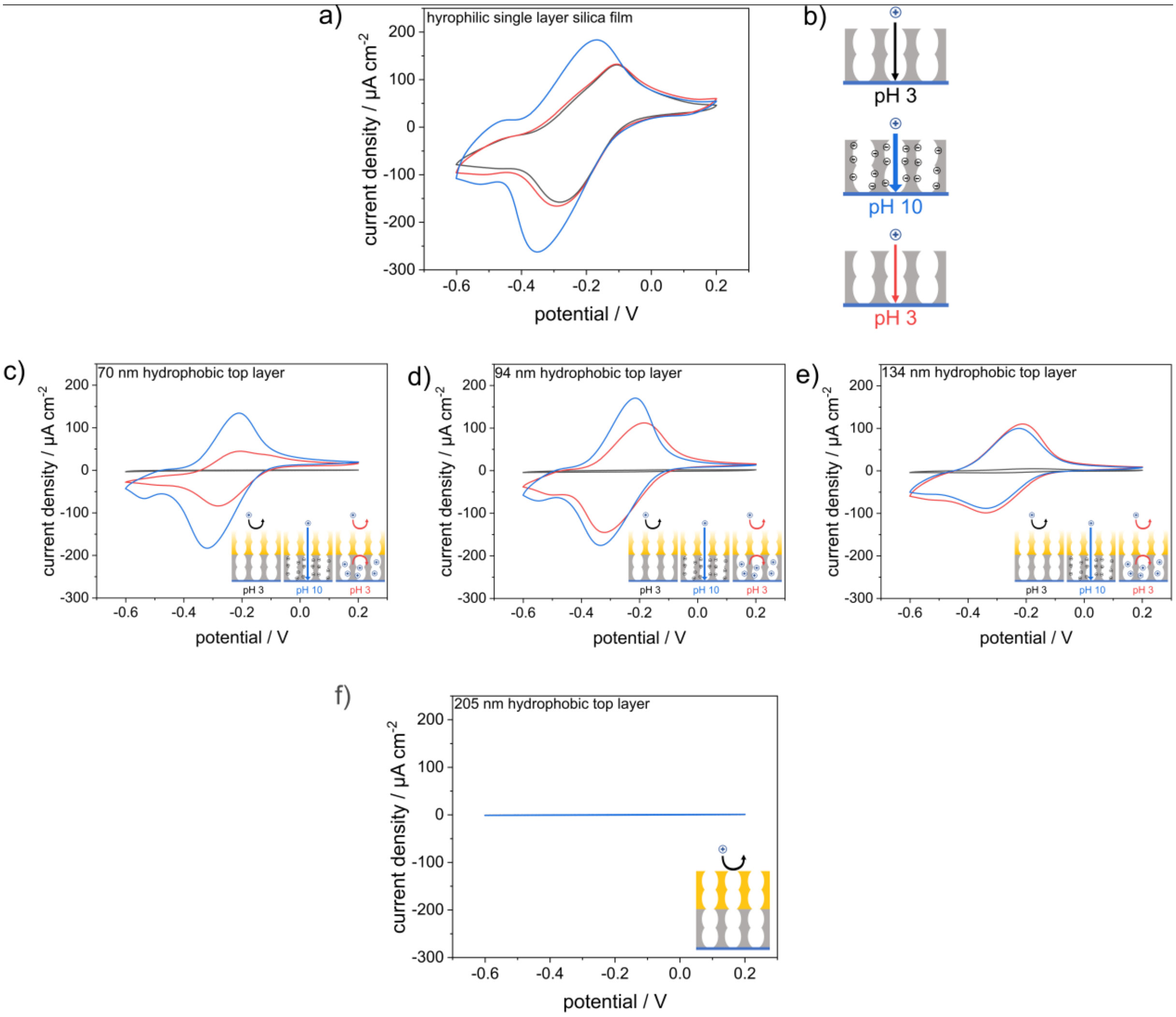

Figure S4: Cyclic voltammograms of a single layer mesoporous silica film (a) and of double layer silica films with a hydrophilic bottom layer and a hydrophobic top layer. The thickness of the bottom layer was held constant whereas the thickness of the top layer was gradually increased from 70 nm (c), 94 nm (d), 134 nm (e) and 205 nm (g), respectively. Schematic views of a single layer hydrophilic silica film (b), double layer silica films with hydrophobic top layer of 70-134 nm in thickness (c, d, e) and a double layer silica film with a hydrophobic top layer of 205 nm in thickness (g) at acidic and basic solution pH with indicated permselectivity of a positively charged probe molecule. Measurements were performed at an acidic solution pH (black), basic solution pH (blue) and again acidic solution pH (red) using 10^{-3} M $[Ru(NH_3)_6]^{2+/3}$+ as probe molecule in 0.1 M aqueous KCl solution. Scanrate 100 mV s^{-1}

To visualize the trapping effect in the absence of an electrode with applied potential, the positively charged fluorophore methylene blue was used. The measuring conditions were kept identical as for CV measurements (1 mM methylene blue in 0,1 M aqueous KCl solution at basic pH) except not applying a potential to an electrode. After CV treatment and extraction in acidic water for 10 min, no fluorophore remained inside the single layer hydrophilic silica film (Figure S5b). Further experiments were done using double layer mesoporous silica films consisting of a hydrophilic bottom layer (100 mol% TEOS, ACA<10°) and a hydrophobic top layer (50 mol% TEOS, ACA~100°). With increasing layer thickness of the hydrophobic top layer, the retention of the fluorophore first increases until a layer thickness of ~80 nm (Figure S5d) before it decreases, which is in accordance with CV measurements (Figure S4). The double layer silica film with a layer thickness of 205 nm show almost no fluorophore residue (Figure S5h).

The darker or brighter ring around the measuring area is an effect of the sealing ring of the CV set-up, that is compressing the silica film.

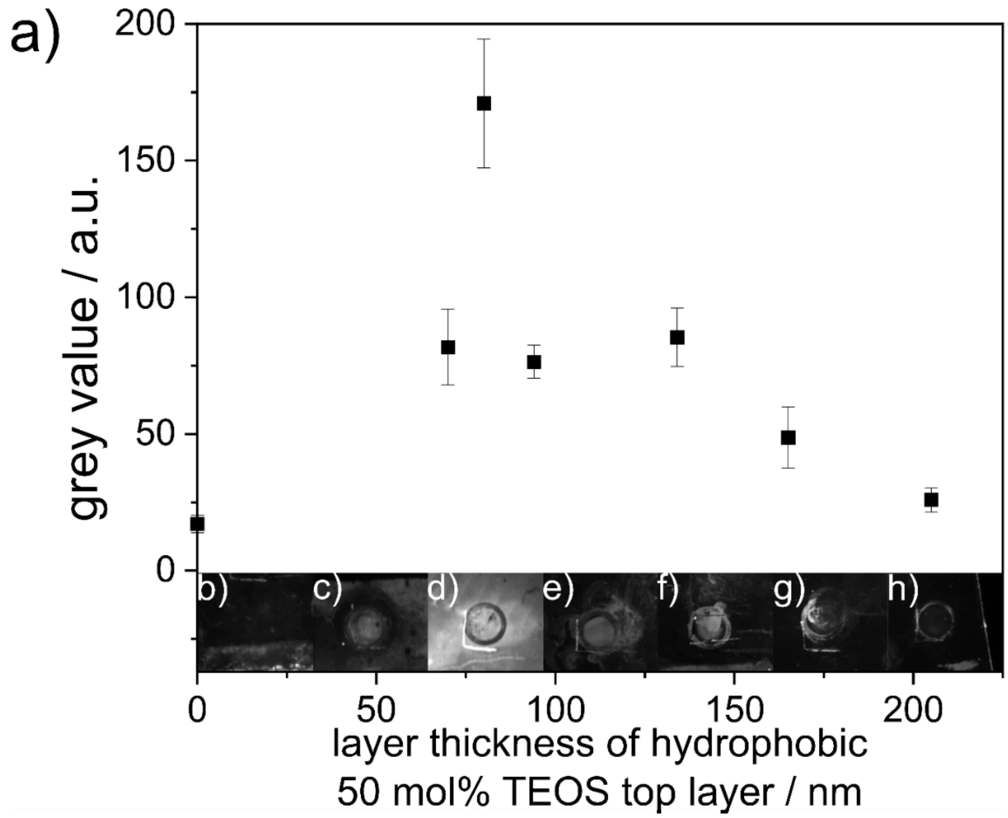

Figure S5: a) Grey value of the image of the measuring area in dependence of the film thickness of the hydrophobic top layer after performing CV measurements using 1mM methylene blue 0.1 M aqueous KCl solution. The film thicknesses of the top layer are 0 nm (b), 70 nm (c), 80 nm (d), 94 nm (e), 134 nm (f), 180 nm (g) and 205 nm (h).

Fluorophore adsorption into double layer silica films with varying hydrophobic top layer thickness

To evaluate the ability of charged probe molecules to overcome a hydrophobic mesoporous silica top layer in a step-gradient wettability mesoporous double layer film, the adsorption of the positively charged fluorophore ATTO647N(-amine) at a basic solution-pH was detected using fluorescence microscopy. Experiments were performed with varying hydrophobic top layer thickness while not using CV and applying a potential. A TIRF setup was used to only observe fluorescence in the lower ~190 nm of the film. The fluorescence intensity was normalized to the background intensity. A very slight increase in fluorescence intensity with increasing hydrophobic top layer thickness up to 134 nm was observed (Figure S6). However, adsorption in all observed samples is very low compared to adsorption of fluorophore in a hydrophilic mesoporous silica film (Figure S2). Mesoporous double layer films with 180-205 nm hydrophobic top layers show no significant fluorophore adsorption. This indicates that without additional external applied forces, adsorption of positively charged probe molecules is strongly hindered by a hydrophobic top layer, depending on the size of the hydrophobic layer.

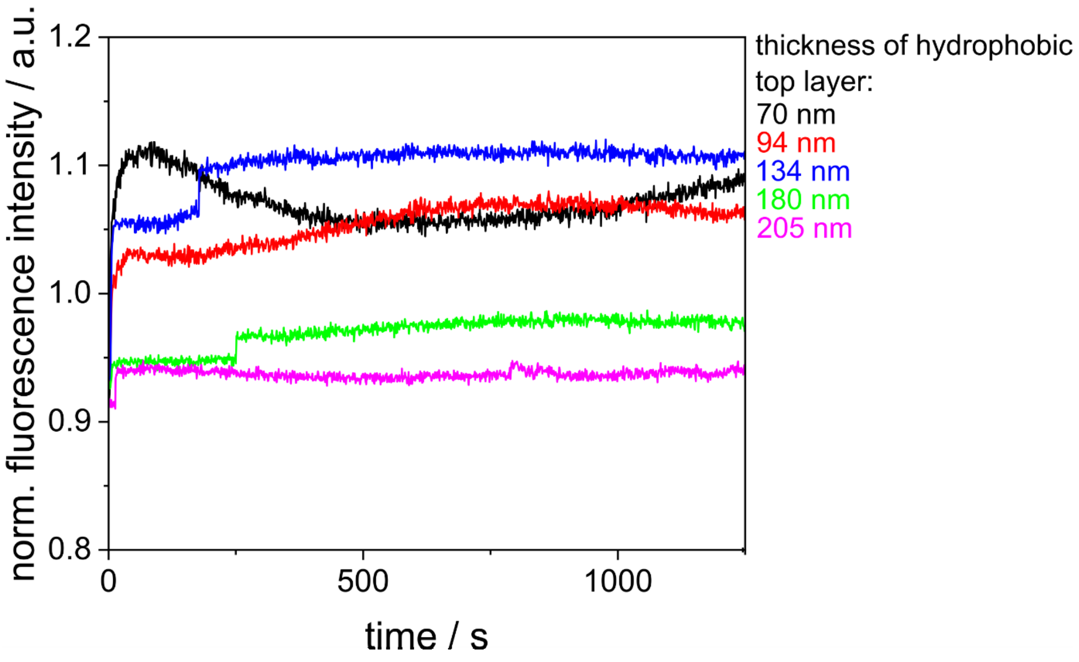

Figure S6: Average fluorescence intensity in the central illuminated region resulting from adsorption of ATTO647N from a 1 µg ml^{-1} aqueous solution (pH 10) into mesoporous silica double layer film with varying thickness of the hydrophobic top layer over time. Intensity was normalized to the background intensity. Illumination was performed using the evanescent field of a TIRF to only excite fluorophore in the lower ~250 nm of the film. Images were acquired with 0.5 fps for 45 min.

References

1. Bruggeman DAG. Berechnung verschiedener physikalischer Konstanten von heterogenen Substanzen. I. Dielektrizitätskonstanten und Leitfähigkeiten der Mischkörper aus isotropen Substanzen. Annalen der Physik. 1935;416(7):636-64.
2. Schubert, U., Sol-Gel-Chemie. *Chemie in unserer Zeit* **2018,** *52* (1), 18-25.
3. Tan, B.; Rankin, S. E., Study of the effects of progressive changes in alkoxysilane structure on sol-gel reactivity. *J Phys Chem B* **2006,** *110* (45), 22353-64.
4. Kenyon IR. The Light Fantastic: A Modern Introduction to Classical and Quantum Optics: Oxford University Press; 2008.
